# Socioeconomic Status Moderates the Impact of Emotional but not Physical Childhood Abuse on Women’s Sleep

**DOI:** 10.1007/s42844-021-00035-9

**Published:** 2021-03-18

**Authors:** Cheryl L. Currie, Erin K. Higa, Lisa-Marie Swanepoel

**Affiliations:** Faculty of Health Sciences, University of Lethbridge, M3083 Markin Hall, 4401 University Drive, Lethbridge, AB T1K 3M4 Canada

**Keywords:** Child abuse, Sleep, Socioeconomic status, Women, Life course

## Abstract

A recent systematic review highlighted associations between childhood abuse and adult sleep quality, and the need for research focused specifically on women and the role of moderating variables. The objectives of the present study were (1) to assess the impact of frequent physical and emotional child abuse on adult sleep among women; and (2) to assess the role that childhood socioeconomic status (SES) could play in moderating these associations. In-person data were collected from women living in a mid-sized city in western Canada in 2019–2020 (*N* = 185; *M age* = 40 years). Sleep quality was measured using the Pittsburgh Sleep Quality Index. Physical and emotional abuse experienced often or very often in childhood were assessed using single items (yes or no). Childhood SES was assessed by a single item and dichotomized at the sample median. Linear regression models examined associations between each form of abuse and continuous adult sleep quality score adjusted for covariates. Statistically significant interactions were stratified and examined by child SES group. Frequent physical and emotional childhood abuse were each associated with clinically and statistically significant increases in past-month sleep problem scores among women in adjusted models. This association was moderated by childhood SES for emotional child abuse, but not physical child abuse. Findings suggest that growing up in an upper-middle to upper SES household may buffer the adverse impact of frequent emotional child abuse on later adult sleep, but may not promote resilience in the context of frequent physical child abuse.

## Introduction

Good sleep is essential for optimal human health and well-being (Buysse et al., 2008). Poor sleep quality is typically defined by subjective experiences such as difficulty falling asleep, frequent nighttime waking, and daytime sleepiness (Krystal & Edinger, 2008). Poor sleep quality is associated with impaired cognitive functioning, increased chronic disease, and all-cause mortality (Miller & Cappuccio, 2007; Knutson et al., 2007; Banks & Dinges, 2007; Walker, 2009; Cappuccio et al., 2010). Poor sleep also impacts mental health including anxiety, distress, and depression (Strine & Chapman, 2005; Wang et al., 2019).

Over the past three decades, the prevalence of sleep disturbance and daytime fatigue have been increasing across studies, suggesting poor sleep quality is on the rise (Hoyos et al., 2015). Sleep problems are more common among women and older adults (Stranges et al., 2012; Madrid-Valero et al., 2017). Proximate behaviours shown to promote good sleep include reducing or eliminating alcohol, nicotine, and caffeine consumption; exercising regularly; maintaining a regular sleep schedule; and mindfulness practices (Irish et al., 2015). Distal exposures may also play a role, with a 2015 systematic review highlighting childhood abuse as a particularly important risk factor for poor adult sleep (Kajeepeta et al., 2015).

Child abuse involves behaviours that cause harm, potential harm, or threats of harm to children under 18 years of age and includes physical, sexual, and emotional abuse (Leeb et al., 2008). Several studies have shown that adults with a history of frequent child abuse are significantly more likely to experience poor sleep as adults (Greenfield et al., 2011; McWhorter et al. 2019). A dose-response association has been observed, with more frequent child abuse resulting in greater sleep disturbance in adulthood (Anda et al., 2006; Greenfield et al., 2011). Reasons for this association are unclear. Studies suggest child abuse can impact brain development and dysregulate the hypothalamic-pituitary-adrenal (HPA) axis in lasting ways that influence sleep (Buckley & Schatzberg, 2005; Mello et al., 2009; Riemann et al., 2010; Gunnar & Vazquez, 2006). Child abuse also alters brain architecture through pathways that can result in greater brain activity during sleep (Heitkemper et al., 2011; Bader et al., 2013).

Despite these findings, most maltreated children appear resilient to sleep problems in adulthood (Walsh et al., 2010; Kajeepeta et al., 2015). Resilience is a dynamic process of positive adaptation and effective coping in the face of adversity (Johnson et al., 2011; Masten & Monn, 2015). Operating on a separate dimension to risk, a resilience factor attenuates the strength of an association between a risk and an outcome (Johnson et al., 2011; Currie et al., 2013). By definition, resilience factors are active in high risk producing conditions such as childhood abuse, acting to reduce the likelihood of a negative outcome (Johnson et al., 2011; Masten & Monn, 2015). Resilience is enhanced by a wide range of individual, family, community, and larger societal factors that benefit well-being and long-term functioning (Masten & Monn, 2015; Southwick et al., 2014; Petersen et al., 2014).

The socioeconomic status (SES) of a household may have a particularly salient impact on resilience given household income influences the resources children have access to, both within and outside the home. This includes the quality of the school they attend, the extracurricular activities they take part in, and the neighbourhood they grow up in; all of which influence the quality of the relationships they have with others outside the home (Cohen et al., 2010; Berger et al., 2009). For decades, studies have demonstrated that children who grow up in high income homes have reduced health-risk behaviours and illness in adulthood, regardless of their later SES as adults (Galobardes et al., 2006; Melchior et al., 2007; Tamayo et al., 2010; Pampel et al., 2011). Building on this body of research, the goal of the present study was to examine the role that child SES could play in moderating the association between childhood abuse and adult sleep among women. 

### Study Objectives

The first objective of this study was to assess the impact of frequent physical and emotional childhood abuse on adult sleep quality among women. Our second objective was to assess the role that childhood SES could play in moderating these associations.

## Methods

### Study Design and Sample

We used data collected from a community-based sample of women living in a mid-sized city in western Canada (population ~100,000) who were enrolled in a randomized controlled trial that examined the impact of wellness interventions on health-risk behaviour (Currie et al., 2019). Ethics approval was granted by the Health Research Ethics Board of Alberta Cancer Committee (ID HREBA.CC-18-0467). All participants provided written informed consent. Participants were eligible if they (1) were 18 years or older; (2) used a nicotine-containing substance, consumed alcohol, or consumed a sugar-sweetened beverage at least once in the past month; (3) lived at a permanent address in the target city; and (4) planned to live in the target city for the next 12 months. Participants were ineligible if they did not meet eligibility criteria, were unwilling to give consent, or lacked capacity to give consent.

### Data Collection

Data were collected between April 2019 and February 2020 from 185 women aged 18 to 88 years who were enrolled in the RCT. All data collected and examined in the present secondary analysis involved survey data collected at baseline before women were randomized to treatment or control. Eligibility screening took place by phone, text, or email as a participant preferred, with data collected within one month of study enrollment. A two-hour in-person data collection appointment then took place with study staff. Participants completed written consent and answered survey questions on an office computer using Qualtrics (Qualtrics XM, USA). A research assistant remained in the room to assist with survey administration and answer participant questions as they completed it. Participants received a $25 honorarium for their time.

### Measures

#### Childhood Abuse

Participants were asked to recall emotional and physical abuse that had occurred before the age of 18. Frequent physical abuse was assessed by the question: “Did a parent or other adult in the household often or very often push, grab, slap, or throw something at you, or ever hit you so hard that you had marks or were injured?” Frequent emotional abuse was assessed by the question: “Did a parent or other adult in the household often or very often swear at you, insult you, put you down, humiliate you, or act in a way that made you afraid that you might be physically hurt?” Response options to each question were yes and no (Felitti et al., 1998).

#### Adult Sleep Quality

The Pittsburgh Sleep Quality Index (PSQI) measured self-reported sleep quality in the past month (Buysse et al., 1989). This 19-item questionnaire measures 7 clinically derived components of sleep, each weighted equally from 0 to 3: subjective sleep quality, sleep latency, sleep duration, sleep efficiency, sleep disturbances, use of sleeping medication, and daytime dysfunction (Buysse et al., 1989). The component scores were added to create a global score of 0–21, with a higher score indicating worse sleep quality (Buysse et al., 1989). A PSQI cut point of 5 has a sensitivity of 90% and a specificity of 87% for identifying cases of sleep disorder (Buysse et al., 1989; Buysse et al., 2008; Backhaus et al., 2002). The internal consistency of the PSQI component score in this study was acceptable (Cronbach’s *α* = 0.66). When comparing sleep quality among women with child abuse histories to controls, little difference has been found between PSQI and polysomnography, which is the gold standard tool for sleep quality measurement (Heitkemper et al., 2011). 

#### Childhood Income Group

Childhood income group was assessed by the question: “If Canadian society were divided into 5 groups, which group do you think you belonged to growing up as a child?” Response options were upper income, upper-middle income, middle income, lower-middle income, and lower income.

#### Covariates

Age, marital status, the number of children participants had, and current adult income group were self-reported (Table 1). Current income group was assessed by the question: “If Canadian society were divided into 5 groups, which group do you think you would currently belong to?” Response options were upper income, upper-middle income, middle income, lower-middle income, and lower income.

**Table 1:** Sample characteristics

Sample characteristics	Total *N* (%)
Total sample	185 (100)
Age	
18–24 years	29 (15.8)
25–34 years	56 (30.4)
35–44 years	27 (14.7)
45–54 years	34 (18.5)
55+ years	38 (20.7)
Married or living common law	
Yes	92 (49.7)
No	93 (50.3)
Do you have children?	
Yes	101 (54.6%)
No	84 (45.4%)
Adult income group	
Upper income	42 (22.7)
Upper-middle income	42 (22.7)
Middle income	78 (42.2)
Low and low-middle income	23 (12.4)
Childhood income group	
Upper income	41 (22.2)
Upper-middle income	46 (24.9)
Middle income	66 (35.6)
Low and low-middle income	32 (17.3)
Childhood abuse	
Frequent emotional abuse	72 (38.9)
Frequent physical abuse	51 (27.6)
Adult sleep quality score (*mean*: 7.3, *SD*: 3.7)	
< 5	44 (23.8)
≥ 5 (poor sleep)	141 (76.2)

### Missing Data

In this sample, 33 of 185 participants (17.8%) chose to not complete all questions relevant to the present analysis. Cases with missing values were not systematically different from cases without missing values across variables. For example, the average age of participants with missing data (39.2 years) and without missing data (40.3 years) was similar . The average adult and childhood household income across participants with missing and non-missing data was “middle income”, and almost half of those with missing data (48%) and without missing data (45%) had children. The Little’s MCAR test chi*-*square of variables examined in our regression models was not significant (chi-square = 7.56, *df* = 9, *p* = 0.58), suggesting the data were missing completely at random (MCAR). However, simulation studies suggest Little’s MCAR test has low statistical power (Enders, 2010). To meet criteria for the use of multiple imputation, the data are required to be MCAR or missing at random (MAR) meaning that the reason for missing data on a variable may be related to some of the observed data, but not related to any of the other missing data. Given most missing data in the study involved the sleep questionnaire (*n* = 25 participants), likely due to its length (19 questions) relative to other measures in the survey (single item measures), we postulate that the missing data was primarily due to the length of the sleep measure and not related to a participant’s choice to skip other questions in the survey, thus meeting the definition for MAR and the use of multiple imputation (Enders, 2017). The Missing Values Analysis extension for SPSS 27.0 was used to conduct multiple imputation. Values were drawn from regression-predicted posterior distributions using 25 iterations. All items from the variables examined in this paper were included in the imputation. Each imputed variable item was constrained to the minimum and maximum range for that item. After imputation, the imputed dataset was analyzed jointly as pooled results per Rubin’s formula (Rubin, 2004).

### Statistical Approach

Measures were summarized with means ±SDs for continuous variables and frequencies for ordinal and dichotomous variables. Separate linear regression models and 95% confidence intervals (CIs) examined associations between each form of child abuse and adult sleep, with sleep quality score examined as a continuous variable. Associations were adjusted for covariates selected a priori from the literature including age, marital status, the number of children a participant had, and their current adult income group (Kajeepeta et al., 2015).

Mental health and health behaviour covariates were not included in models given the impacts of childhood trauma on mental health and behaviour are well documented across studies (Afifi et al., 2014; Currie, 2006; Currie & Tough, 2021; Petersen et al., 2014). Thus, controlling for mental health variables, which likely sit on the causal pathway between child abuse and adult sleep, would result in biased estimates. Controlling for such intermediate variables introduces bias by decomposing the total effect of x on y into its parts (Rohrer, 2018; Achen, 2005). If that is the goal, one should conduct a mediation analysis using appropriate techniques such as the cross products of coefficients method, which was beyond the scope of this paper (Hayes, 2017).

To examine multicollinearity, variance inflations factors (VIFs) were calculated across all variables included in regression models. All VIFs were under the threshold of 10 (range: 0.02 to 6.11), indicating multicollinearity was not a significant concern. To examine the role that childhood income might play in buffering the impacts of child abuse on adult sleep, multiplicative interaction terms were created by forming a product term between each form of abuse and childhood income group and calculating two *R*^2^ values, one for the main-effects-only model, and another with the product term added, with the interaction deemed present if the difference between the two *R*^2^ values was statistically significant as determined by an *F*-statistic. If significant, the sample was stratified into lower and higher childhood income groups using the sample median for this variable. This resulted in 52% of the sample represented in a low to middle childhood income group (*n* = 97), and 48% of the sample represented in an upper-middle to upper childhood income group (*n* = 88). Linear regression models and 95% CIs were then used to examine associations between child abuse and adult sleep for women in each of the two child income categories adjusting for age, marital status, number of children, and current adult income group (Szklo & Nieto, 2018). To provide additional information, given abuse types often overlap in childhood, we also conducted a supplementary analysis to examine the impact of emotional child abuse on adult sleep in the subsample that reported no physical child abuse (*n* = 134). All data were analyzed using IBM SPSS 27.

## Results

### Description of the Sample

The mean age was 40.1 years (*SD* = 14.7, *range* 18–88 years). As shown in Table 1, approximately half the sampe were married or living common-law. Participants had an average of 1.3 children (*SD* = 1.3, *range* 0 to 5). Most women identified as middle income or greater in adulthood (87.4%), and in childhood (82.6%). The association between childhood and adult income group was weak and statistically significant (*Pearson’s r* = 0.19, *p* = 0.01). The average age of women raised in low to middle income homes (39.3 years) was not significantly different than women raised in upper-middle and upper income homes (41.1 years; *Independent Samples t-test* = 0.86, *p* = 0.39). The number of children participants had also did not vary based on whether they grew up in low to middle income houseolds (1.2 current children) or upper-middle and upper income homes (1.3 current children; *Independent Samples t-test* = 0.76, *p* = 0.45). In terms of marital status, 53% of women who grew up in low and middle income households were currently married or living common-law, compared to 47% of women who grew up in upper-middle and upper income households; this difference was not statistically significant (*Phi coefficient*= 0.06, *p* = 0.42).

### Adult Sleep

Most women  reported some sleep problems in the past month, with 75.7% scoring 5 or higher on the PSQI measure. The average sleep quality score for the sample was 7.3 (*SD* 3.6, *range* 1 to 18). The mean difference in sleep score between women raised in low to middle (*Mean* 6.8, *SD* 3.7) and upper-middle to upper income homes (*Mean* 7.9, *SD* 3.2) approached statistical significance(*Independent Samples t-test* = 1.77, *p* = 0.08).

### Childhood Abuse

Approximately 4 in 10 women (41.1%) experienced frequent physical or emotional abuse in childhood. Approximately one quarter (24.3%) had experienced physical abuse, meaning that they were often or very often pushed, grabbed, slapped, had objects thrown at them, and/or hit hard enough to create marks or injury by adult caregivers when they were children . Frequent emotional abuse was reported by 36.2% of women, meaning that they often or very often experienced adult caregivers swearing at them, insulting them, putting them down, humiliating them, or acting in ways that made them feel they could be physically hurt as children. The association between physical and emotional childhood abuse was strong and significant (*Phi coefficient *= 0.70, *p* = 0.001). Only 5.9% of women who reported physical child abuse did not report emotional child abuse. About one-third who reported emotional child abuse did not report physical child abuse (32.8%).

### Physical Child Abuse and Adult Sleep

Women who experienced frequent physical child abuse had a sleep problem score that was 1.8 points higher than women who did not in a linear regression model adjusting for covariates (Table 2, Model 1). In Model 2, a multiplicative interaction term for physical child abuse and childhood income was added. The product term was not statistically significant. A hierarchical *F-statistic* comparing the *R*^2^ values for the main-effects-only model and Model 2 with the product term added was not statistically significant (*F-Change statistic* = 1.71, *df* = 177, *p* = 0.26), indicating no interaction between physical abuse and SES in childhood.

**Table 2:** Linear regression models for the direct effects of physical childhood abuse on adult sleep score*

	*N*	*B* (95% CI)	*SE*	*p*
Model 1: Full sample	185			
Physical childhood abuse		**1.82 (0.53, 3.11)**	**0.66**	**0.006**
Age		0.02 (−0.02, 0.06)	0.02	0.33
Marital status		0.21 (−0.97, 1.38)	0.60	0.73
Number of children		0.36 (−0.83, 0.11)	0.24	0.14
Adult income group		0.44 (−0.12, 1.00)	0.29	0.12
Model 2: Interaction	185			
Physical childhood abuse		3.72 (−0.45, 7.89)	2.12	0.08
Age		0.02 (−0.03, 0.06)	0.02	0.40
Marital status		0.10 (−1.09, 1.29)	0.61	0.87
Number of children		0.37 (−0.84, 0.10)	0.24	0.12
Adult income group		0.40 (−0.16, 0.10)	0.29	0.16
Child income group		1.03 (−0.53, 2.59)	0.79	0.20
Physical childhood abuse x child income		−0.54 (−1.63, 0.55)	0.56	0.33

*Statistically significant variables presented in bold. *B*, unstandardized beta weight

### Emotional Child Abuse and Adult Sleep

Women who experienced frequent emotional child abuse had a sleep problem score that was 1.7 points higher than women who did not in a linear regression model adjusting for covariates (Table 3, Model 1). In Model 2, a multiplicative interaction term for emotional child abuse and childhood income was added to the model. The product term was statistically significant. A hierarchical *F-statistic* comparing the *R*^2^ values for the main-effects-only model and Model 2 with the product term added was significant (*F-Change statistic* = 3.41, *df* = 177, *p* = 0.05). Given this, the sample was stratified into two child income groups using the sample median. As shown in Table 3 (Model 3), among women who grew up in low and middle income households, frequent emotional child abuse was associated with a 2.6-point increase in adult sleep problems in the past month adjusting for covariates. Among women who grew up in middle-upper to upper income households, frequent emotional child abuse was not significantly associated with adult sleep problems.

**Table 3:** Linear regression models for the direct effects of emotional childhood abuse on adult sleep score*

	*N*	*B* (95% CI)	*SE*	*p*
Model 1: Full sample	185			
Emotional childhood abuse		**1.67 (0.53, 3.11)**	**0.61**	**0.006**
Age		0.02 (−0.02, 0.06)	0.02	0.38
Marital status		0.18 (−0.97, 1.38)	0.60	0.77
Number of children		0.32 (−0.83, 0.11)	0.24	0.18
Adult income group		0.32 (−0.12, 1.00)	0.30	0.28
Model 2: Interaction	185			
Emotional childhood abuse		**5.53 (1.59, 9.48)**	**2.12**	**0.006**
Age		0.02 (−0.03, 0.06)	0.02	0.41
Marital status		0.09 (−1.07, 1.25)	0.60	0.88
Number of children		0.37 (−0.83, 0.09)	0.24	0.12
Adult income group		0.31 (−0.27, 0.89)	0.30	0.30
Child income group		**1.81 (0.29, 3.33)**	**0.78**	**0.02**
Emotional childhood abuse x child income		−**1.10 (**−**2.12,** −**0.08)**	**0.52**	**0.04**
Model 3: Low to middle childhood income group	97			
Emotional childhood abuse		**2.59 (0.80, 4.38)**	**0.91**	**0.005**
Age		0.02 (−0.08, 0.04)	0.03	0.51
Marital status		0.06 (−1.60, 1.73)	0.85	0.94
Number of children		0.30 (−0.92, 0.31)	0.31	0.33
Adult income group		0.14 (−0.66, 0.93)	0.41	0.74
Model 4: Middle-upper to upper childhood income group	88			
Emotional childhood abuse		0.35 (−1.20, 1.91)	0.79	0.65
Age		**0.07 (0.01, 0.14)**	**0.03**	**0.02**
Marital status		0.01(−1.57, 1.60)	0.81	0.98
Number of children		0.45 (−1.16, 0.26)	0.36	0.22
Adult income group		0.47 (−0.34, 1.28)	0.41	0.26

*Statistically significant variables presented in bold. *B*, unstandardized beta weight.

In Table 4 we present a reanalysis of the findings for emotional child abuse, excluding women who had experienced physical child abuse in the sample. The purpose of this subanalysis was to understand if emotional abuse alone might impact adult sleep for women, and the role that childhood income could play in this association. Within this subsample (*n* = 134) who had not experienced physical child abuse, 24 women reported emotional abuse in childhood (17.9%). When the multiplicative interaction term for emotional child abuse and childhood income was added, it was statistically significant. A hierarchical *F-statistic* comparing the *R*^2^ values for the main-effects-only analysis in Model 1 with Model 2 that included the product term was statistically significant (*F-Change statistic* = 4.67, *df* = 126, *p* = 0.02). Thus, we stratified the sample into two child income groups.

**Table 4:** Linear regression models for the direct effects of emotional childhood abuse on adult sleep score among women who did not experience physical abuse in childhood*

	*N*	*B* (95% CI)	*SE*	*p*
Model 1: Full sample	134			
Emotional childhood abuse		0.59 (−1.05, 2.23)	0.84	0.48
Age		0.04 (−0.01, 0.09)	0.02	0.11
Marital status		0.02 (−1.28, 1.30)	0.66	0.98
Number of children		**0.58 (0.03, 1.13)**	**0.28**	**0.04**
Adult income group		**0.69 (0.01, 1.37)**	**0.35**	**0.05**
Model 2: Interaction	134			
Emotional childhood abuse		**9.53 (1.53, 17.54)**	**4.08**	**0.02**
Age		0.04 (−0.01, 0.12)	0.02	0.10
Marital status		0.01 (−1.28, 1.30)	0.66	0.98
Number of children		**0.65 (0.11, 1.18)**	**0.27**	**0.02**
Adult income group		0.58 (−0.09, 1.25)	0.34	0.09
Child income group		**3.03 (0.08, 5.25)**	**1.13**	**0.007**
Emotional childhood abuse x child income		−**2.33 (**−**4.31,** −**0.35)**	**1.01**	**0.02**
Model 3: Low to middle childhood income group	24			
Emotional childhood abuse		**2.77 (0.01, 5.53)**	**1.41**	**0.05**
Age		0.01 (−0.05, 0.08)	0.03	0.69
Marital status		−0.06 (−1.79, 1.68)	0.89	0.95
Number of children		0.64 (−0.04, 1.33)	0.35	0.06
Adult income group		0.47 (−0.42, 1.35)	0.45	0.30
Model 4: Middle-upper to upper childhood income group	110			
Emotional childhood abuse		0.35 (−1.20, 1.91)	0.79	0.65
Age		**0.07 (0.01, 0.14)**	**0.03**	**0.02**
Marital status		0.01(−1.57, 1.60)	0.81	0.98
Number of children		0.45 (−0.26, 1.16)	0.36	0.22
Adult income group		0.47 (−0.34, 1.28)	0.41	0.26

*Statistically significant variables presented in bold. *B*, unstandardized beta weight

As shown in Table 4 (Model 3), among women who grew up in low and middle income households, frequent emotional child abuse was associated with 2.8-point increase in adult sleep problems adjusting for covariates, which was similar to the impact of emotional child abuse on adult sleep problems among women who may have also experienced physical abuse (2.6-point increase in sleep problem score). It is notable that this association was statistically significant given it was likely underpowered due to the small sample size (*n* = 24). Among women who grew up in middle-upper to upper income households, frequent emotional child abuse was not associated with adult sleep among women who had not experienced physical abuse.

## Discussion

Frequent emotional and physical childhood abuse were each associated with reduced adult sleep quality among women. When the sample was examined as a whole, sleep problem scores were 1.7 and 1.8 points higher among women who had experienced emotional and physical child abuse; respectively. These increases are clinically relevant given a score of 5 on the sleep measure used (PSQI) has been shown to identify cases of sleep disorder with a sensitivity of 90% (Buysse et al., 1989). In keeping with other studies, almost all women who reported physical child abuse also reported emotional child abuse in this study (Chamberland et al., 2011). Given this overlap, the associations observed likely represent the combined impact of both physical and emotional abuse on adult sleep for some participants. When women who had experienced physical child abuse were removed from the sample, emotional child abuse was not significantly associated with adult sleep before taking childhood SES into account.

### Emotional Child Abuse and Adult Sleep: Moderation by Childhood SES

The present findings suggest childhood SES may play a role in buffering the impact of emotional child abuse on adult sleep. Among women who grew up in *low to middle income* homes, frequent emotional child abuse was associated with an almost 3-point increase in adult sleep problems after adjustment for covariates including adult SES. This association changed little when women who had also experienced physical child abuse were excluded from the analysis. In contrast, frequent emotional child abuse was not associated with adult sleep among women who grew up in *high income* homes. While we did not have a sample size sufficient to examine mediators of this moderation, hypotheses can be put forward.

It is well documented that children who grow up in high SES households are more likely to be exposed to resource-rich environments both within and outside the home that help promote the development of emotional regulation, positive mental health, and positive health behaviour (McEwen & Gianaros, 2010; Southwick et al., 2014; Postilnik & Howett, 2020; Pampel et al., 2011). Link and Phelan (1995) have argued that high SES groups, including children, have access to resources that are so extensive as to make SES a fundamental *cause* of their good health and well-being (Link & Phelan, 1995). Applying this literature to the present findings, it may be theorized that women who grew up in high income homes that were emotionally abusive had greater access to resources outside the home that could confer resilience, compared to women who grew up in low and middle income homes. For example, high income families have increased means to involve children in extracurricular activities. Such activities have been shown to reduce the harms of parental emotional abuse by connecting children to caring adults and activities that buffer against these at-home difficulties (CDC, 2019; Williams, 2019). An important avenue for future research would be to examine the role that differential access to childhood resources outside the home among high vs. low and middle SES children could play in reducing the impact of emotional child abuse on adult sleep and other adverse outcomes (e.g. mental health).

### Physical Child Abuse and Adult Sleep: No Moderation by Childhood SES

Research suggests SES may be less effective in conferring resilience in some situations compared to others (Pampel et al., 2011). This was observed in the present study. We found childhood SES did not confer resilience against the adverse impact of frequent physical child abuse on adult sleep. A large body of evidence has shown that frequent child abuse can result in significant changes in a child’s developing brain (De Bellis & Zisk, 2014; Danese & McEwen, 2012; Norman et al., 2012). These changes alter hyperarousal in the face of stress (fight or flight) and can result in entrenched beliefs that survival is in jeopardy, a sense of hopelessness and helplessness, and other adverse mental health states that impact sleep both in childhood and later adulthood (De Bellis & Zisk, 2014; Danese & McEwen, 2012; Norman et al., 2012; Spilbury, 2009; Siltala et al., 2019; Hamilton et al., 2018; Brindle et al., 2018; Park et al., 2020). The impact of childhood trauma on the developing brain and the sequalae resulting from these changes (e.g. HPA axis dysregulation, adverse mental health states) are important to consider given trauma exposure before 18 years of age has been shown to impact adult actigraphy-assessed sleep health, while trauma exposure after 18 years of age has not (Brindle et al., 2018).

The reasons why a high childhood SES could not buffer the impacts of physical child abuse on adult sleep in the present study cannot be ascertained from the results. However, research has shown that physical child abuse has more consequential impacts on the developing brain than emotional abuse (Kuhlman et al., 2015). Thus, it is biologically plausible that frequent physical child abuse may impact adult sleep through altered brain pathways that are significant enough that they cannot be circumvented by a resource-rich high SES environment. Future studies with larger samples are recommended to build on this work through moderated-mediation analyses that test potential causal mechanisms behind the interactions observed.

Strengths of this study include the use of a community-based sample of women which adds to the literature on gender, child abuse, and adult sleep (Fisher et al., 2009; Koskenvuo et al., 2010). Other strengths include the use of a validated measure of sleep and an examination of the role that childhood SES could play in moderating the impact of child abuse on adult sleep, which is novel in the literature.

Study limitations include a cross-sectional study design and a convenience sample which precludes inferences about causal inference. Given this study was a secondary analysis, a sample size calculation was not conducted for the variables examined. While recruitment materials for the present study did not mention child abuse, all women in the sample used a nicotine-containing substance, consumed alcohol, or consumed a sugar-sweetened beverage in the past month. Smoking and alcohol use are elevated among women who have experienced child abuse, which may have resulted in elevated childhood abuse levels in this study (Felitti et al., 1998). Overall, 36% and 24% of women in this study reported emotional and physical childhood abuse, respectively. These results are similar to a 2011–2014 surveillance study across 23 US states in which 34% and 18% of women reported emotional and physical childhood abuse, respectively (Merrick et al., 2018). Similarly, a large study conducted in the province that the present study took place (Alberta) found 36% and 17% of adult women reported emotional and physical child abuse (Currie et al., 2020). We also note that the prevalence of physical child abuse in the present study was 4% lower than the Wave II CDC-Kaiser ACE Study with 3500 women in California, suggesting physical child abuse varies across place and time and may not be unusually high in the present sample (Merrick et al., 2017).

Although sleep problems were common in this study, with 76% of the sample having a PSQI sleep scores ≥5, women experience a high burden of sleep problems compared to men. Our findings were similar to a 2017 study that found 73% of community-based women had PSQI scores ≥5 (Rai & Sherkhane, 2017). It is possible that the COVID-19 pandemic impacted sleep among participants in this study. However,  our data collection ended in February 2020 which was one month before the first case of COVID-19 in Alberta, and well before public restrictions were imposed. That said, it is possible that news reports about the pandemic in other regions adversely impacted sleep in this study. We also note that all women in our sample had used a nicotine-containing substance, consumed alcohol, or consumed a sugar-sweetened beverage in the past month. Studies have shown that these behaviours can reduce sleep quality among women (Irish et al., 2015). Results may also be limited by recall bias and/or social desirability bias as some women may have been hesitant to report child abuse or belonging to low income groups. That said, retrospective reports of major, easily defined ACEs have acceptable psychometric properties (Reuben et al., 2016), and a recent systematic review concluded that retrospective measures of childhood trauma have strong sensitivity in terms of detecting true cases (Baldwin et al., 2019). However, we cannot rule out that low income may have been underreported, and that disrupted sleep patterns may have impaired memory consolidation for some participants (Rasch & Born, 2013).

In terms of the generalizability of the present findings to men, research examining gender-based differences in the association between child abuse and adult sleep has been inconclusive (Kajeepeta et al., 2015). Multiple studies have shown that childhood abuse is more prevalent among women and that women may be more likely to experience adverse outcomes from child abuse than men (Fisher et al., 2009; Haatainen et al., 2003; Ramsawh, Ancoli-Israel, Sullivan, Hitchcock, & Stein, 2011). It is also possible that the role that childhood SES could play in buffering the impacts of child abuse on adult sleep may differ for men. Thus, we believe it would be premature to generalize the interactions observed  in this study to men, and recommend additional studies to examine these associations. 

## Conclusions

Women experience a high burden of sleep problems (Madrid-Valero et al., 2017). Previous research suggests child abuse may play a role. The present findings suggest a high childhood SES may buffer the impacts of frequent emotional child abuse on adult sleep. However, a high childhood SES did not buffer the adverse impacts of frequent physical child abuse on adult sleep among women in this study.
